# Evaluation of *Toxoplasma gondii *as a live vaccine vector in susceptible and resistant hosts

**DOI:** 10.1186/1756-3305-4-168

**Published:** 2011-08-28

**Authors:** Jun Zou, Xiao-Xi Huang, Guang-Wen Yin, Ye Ding, Xian-Yong Liu, Heng Wang, Qi-Jun Chen, Xun Suo

**Affiliations:** 1 National Animal Protozoa Laboratory, College of Veterinary Medicine, China Agricultural University, Beijing, 100193, China; 2 Department of Veterinary Pathology, College of Veterinary Medicine, China Agricultural University, Beijing, 100193, China; 3 Department of Etiology, Molecular Parasitology Laboratory, Institute of Basic Medical Sciences, Chinese Academy of Medical Sciences and Peking Union Medical College, Beijing 100005, China; 4 Institute of Pathogen Biology, Chinese Academy of Medical Sciences, Dong Dan San Tiao 9, Beijing 100730, China

## Abstract

**Background:**

*Toxoplasma gondii *has been shown to trigger strong cellular immune responses to heterologous antigens expressed by the parasite in the inbred mouse model [1]. We studied the immune response induced by *T. gondii *as an effective vaccine vector in chickens and rabbits.

**Results:**

*T. gondii *RH strain was engineered to express the yellow fluorescent protein (YFP) in the cytoplasm. A subcutaneous injection of the transgenic *T. gondii *YFP in chickens afforded partial protection against the infection of transgenic *E. tenella *YFP. *T. gondii *YFP induced low levels of antibodies to YFP in chickens, suggesting that YFP specific cellular immune response was probably responsible for the protective immunity against *E. tenella *YFP infection. The measurement of T-cell response and IFN-γ production further confirmed that YFP specific Th1 mediated immune response was induced by *T. gondii *YFP in immunized chickens. The transgenic *T. gondii *stimulated significantly higher YFP specific IgG titers in rabbits than in chickens, suggesting greater immunogenicity in a *T. gondii *susceptible species than in a resistant species. Priming with *T. gondii *YFP and boosting with the recombinant YFP can induce a strong anti-YFP antibody response in both animal species.

**Conclusions:**

Our findings suggest that *T. gondii *can be used as an effective vaccine vector and future research should focus on exploring avirulent no cyst-forming strains of *T. gondii *as a live vaccine vector in animals.

## Background

A variety of viruses and bacteria have been used successfully as live vaccine vectors [2-6]. The antigen delivering efficiency and the type of immune response of live vaccine vectors depends on their replication at infected sites and in target cells [7]. An effective live vaccine vector should have the capacity to infect a wide range of target cells with high efficiency and present effectively heterologous antigens to T cells. In addition, a live vaccine vector should also satisfy the requirement of safety and the ease of transfection of foreign DNA into the vector [8].

*Toxoplasma gondii *is an obligate intracellular parasite. It can infect any nucleated cells of warm-blood vertebrates [9-12] and induce strong humoral, mucosal and cellular immune responses, making it an attractive system for delivering heterologous antigens [9]. Avirulent strains of *T. gondii *have been tested to immunize livestock and studied in experimental animals to prevent congenital toxoplasmosis [13]. A commercial live S48 strain vaccine (Ovilis. Toxovax^®^) for veterinary use has already been approved in some countries [14-16]. Because of the strong immunogenicity, availability of avirulent strains and the ease of genetically engineering stable parasite lines, *T. gondii *has the potential to be explored as a live vaccine vector for bacterial, viral and parasite pathogens [17].

Studies on the immune response to *T. gondii *infection have been conducted extensively in the mouse [1,18,19]. Green fluorescent protein (GFP) has been extensively utilized as the reporter protein in genetic manipulation [20-22], and it was also used as a model antigen to study the antigen delivery to target the specific immune response pathway [23]. We posed the following questions: (-) Could foreign antigens expressed by *T. gondii *stimulate antigen-specific protective immune responses in chickens; (-) whether there is any difference in antigen specific immune responses induced by transgenic *T. gondii *in chickens, which are naturally resistant to *T. gondii *infection, and rabbits, which are susceptible to *T. gondii *infection.

In this study, we developed a transgenic *T. gondii *that expressed the yellow fluorescent protein (YFP), a yellow version of GFP [24], as a model antigen. We firstly demonstrated that the transgenic *T. gondii *YFP elicited YFP-specific immune responses that conferred partial protection against a challenge with YFP-expressing *E. tenella*. We also showed that immunization with transgenic *T. gondii *YFP induced greater YFP-specific humoral immune responses in rabbits than in chickens. Our data have obvious implications on the utilization of *T. gondii *or other apicomplexa protozoa as a live vaccine vector. A commercial live vaccine strain S48 or avirulent no cyst-forming strains of *T. gondii *need to be used to explore *T. gondii *as a live vaccine vector in animals in the future study.

## Materials and methods

### Parasite

The wild type RH strain of *T. gondii *and its stably transfected line were maintained by serial passages in African green monkey kidney (VERO) (Shanghai Institutes For Biological sciences, CAS) cells in DMEM supplemented with FBS (10% v/v), penicillin (200 U ml^-1^) and streptomycin (20 mg ml^-1^) in a humidified atmosphere of 5% CO_2 _at 37°C. Stable YFP-transfected *Eimeria tenella *(*E. tenella *YFP) was constructed, maintained and propagated in coccidia-free 4-day-old AA broilers [25], briefly YFP expression vector was transfected into the wild type *E. tenella *sporozoites, and the transfected sporozoites were inoculated into chickens. At 6-9 days post-infection, oocysts were collected from feces of chickens according to procedures described previously [26]. The YFP positive oocysts were sorted by a MoFloTM cell sorter (Dako Cytomation, Denmark) four times until the percentage of fluorescent oocysts reached 90%.

### Plasmid construct

**Figure 1:**
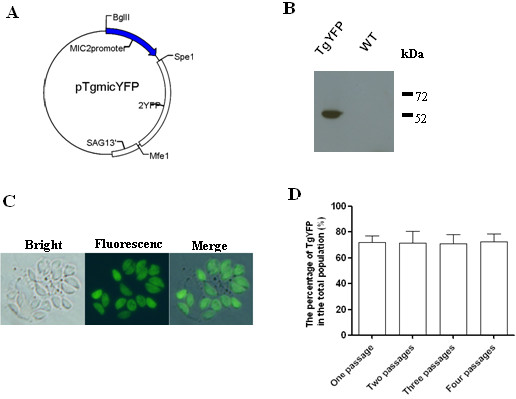
**Expression of yellow fluorescent protein (YFP) by *T. gondii *transfected with the pTgmicYFP plasmid**. A, Plasmid map of pTgmicYFP. B, Western blot of YFP expressed by the transgenic *T. gondii YFP *(TgYFP) and wild type *T. gondii *(WT) using the rabbit anti-GFP antisera and a sheep anti-rabbit IgG HRP-conjugate. C, *T. gondii *YFP in murine macrophages observed by fluorescence microscopy, confirming the location of YFP by *T. gondii *YFP. D, Fitness of *T. gondii *YFP and wild type *T. gondii *at a ratio of 7:3 in mice.

The pTgmicYFP plasmid was constructed from the pTgsagYFP, which was previously constructed in our laboratory [27]. The sag1 promoter of *T. gondii *was replaced by the *T. gondi *microneme 2 (MIC2) promoter (1.48 kb) before the insertion of the YFP reporter gene within the 5' sequence of MIC2 and 3' sequence of SAG1 of *T. gondii *(Figure 1A).

### Transgenic *T. gondii*

The RH strain *T. gondii *tachyzoites were propagated, harvested and purified according to established protocols [28]. Freshly purified tachyzoites were suspended in the complete cytomix buffer to a final concentration of 5 × 10^7 ^ml^-1^. The pTgmicYFP plasmid was linearized with the restriction endonuclease BglII and transfected to the parasites by electroporation [29]. Fifty-150 μl linearized pTgmicYFP, 100 U BglII and 1 × 10^7 ^tachyzoites in a 4 mm cuvette were subjected to electroporation (Gene Pulser II™, Bio-Rad, USA) at a peak voltage of 2 kV and a capacitance of 25 μF. After electroporation, the parasites were left undisturbed at room temperature for 20 min and then inoculated onto confluent MDBK or VERO cells (Shanghai Institutes For Biological sciences, CAS) in the modified DMEM medium.

The transfected tachyzoites were released and sorted by a MoFlo™cell sorter (Dako Cytomation, Denmark), and the sorted tachyzoites were cultured again in MDBK or VERO cells. After about five passage-sorting cycles, the transgenic line, named as *T. gondii *YFP was cloned by limiting dilution in 96-well plates [28].

YFP expression in the transfected tachyzoites was verified by Western blotting. The transgenic and wild parasites were harvested from VERO cells, lysed in SDS sample buffer, and boiled for 10 min. The YFP protein was identified by Western blotting using rabbit anti-GFP antisera (Proteintech, China), goat anti-rabbit IgG conjugated to alkaline phosphatase (Proteintech, China) and chemiluminescent detection (CWBIO, China).

YFP expression by the transgenic line *in vivo *was studied in mice. The transgenic *T. gondii *YFP was propogated in Balb/c mice by i.p. inoculation with 1 × 10^4 ^tachyzoites per animal. Replicating *T. gondii *YFP tachyzoites in murine macrophages were harvested from the peritoneal cavity with 4 ml of sterile saline 3 days after inoculation. Parasites in intact macrophages were examined by fluorescence microscopy with 488 nm excitation and 508 nm emission filters.

To determine the fitness of *T. gondii *YFP, the transgenic and wild type *T. gondii *at a ratio of 7:3 were inoculated i.p. to mice at 10^4^/animal. The proportion of *T. gondii *YFP, which were harvested from the peritoneal cavity 3-5 days after inoculation, was measured by FACS after each passage in mice.

### Tachyzoite antigens preparation and expression of YFP proteins in bacteria

A crude extract of proteins was obtained by repeated freezing and thawing of 2 × 10^8 ^RH tachyzoites in liquid nitrogen. The lysates were centrifuged at 5000 × g for 20 min at 4°C and the supernatants were collected. The YFP gene was cloned from the pTgtubNP-YFP/sagCAT vector [30]. The cloned gene was inserted into the pET-21a vector (Novatech, France). The resulting plasmid was transformed to *E. coli *BL21 (DE3) cells (Transgen Company, China) with ampicillin selection. The recombinants were harvested after 6 h of induction with IPTG (Isopropyl β-D-1-Thiogalactopyranoside). The YFP protein was purified using a His bind buffer kit (Novatech, France).

### Animals and immunization

AA broiler chickens were separately caged in an air-conditioned room. The 15-day-old chickens (n = 10) were immunized s.c. or i.m. with the transgenic or wild type *T. gondii *(5 × 10^6^/chicken) in complete cytomix buffer (CCB), 160 μg recombinant YFP emulsified in Freund complete adjuvant (FCA) or CCB. Female New Zealand white rabbits (90 days of age) were inoculated with 1 × 10^7 ^parasites. The immunized or non-immunized chickens and rabbits were boosted i.m. with 160 μg (chicken) or 500 μg (rabbit) YFP emulsified with FCA. The booster dose was administered 20 (chicken) or 35 (rabbit) days after the primary immunization. Serum anti-YFP antibodies were determined by ELISA 10 days after the primary immunization or the booster immunization.

Additional groups of 10 day old white Leghorn chickens (n = 10) were immunized s.c. twice with the transgenic or wild type *T. gondii *tachyzoites or160 μg recombinant YFP emulsified in Freund complete adjuvant (FCA) or complete cytomix buffer (un-immunized control) at a dose interval of 15 days. The primary immunization tachyzoites was 5 × 10^6^, and the booster dose was 1 × 10^7^. All chickens were challenged orally with transgenic *E. tenella *YFP oocysts (1 × 10^3^) 15 days after the booster immunization dose. The immune protection of *T. gondii *YFP against *E. tenella *YFP, which carry the same model antigen, was assessed by fecal oocyst excretion and cecum lesions 9 days after the challenge.

### Determination of antibody titers

Serum antigen specific IgG of the immunized chickens or rabbits was measured by ELISA. A 96-well microtiter plates were coated with the recombinant YFP harvested from *E. coli BL21 *bacteria (described above) at 2 μg/ml or tachyzoite antigens at 5 μg/ml in 0.05 M bicarbonate buffer (pH 9.6) overnight at 4°C, and blocked for 2 h at 37°C with 5% milk powder (Difco™skim milk, BD) in PBST(PBS containing 0.05% Tween 20)before washing with PBST. Serially diluted serum samples were added and incubated for 1 h at 37°C. Antigen-specific antibodies were detected with HRP-labeled anti-chicken IgG with 1:1000 dilution or anti-rabbit IgG with 1:5000 dilution in 2% milk-PBST for 1 h at 37°C. The ELISA was developed with 100 μl/well of 10 mg/ml TMB solution in 0.025 M phosphate-citrate buffer (Sigma). The reaction was stopped by 0.2 M H_2_SO_4 _and the optical density (OD) at 450 and 630 nm was measured by a plate reader (Bio-Rad, CA, USA). The serum antibody titer was defined as the highest dilution that gave a test/naïve serum OD ratio of 2.1 or higher.

### Real-time RT-PCR

Three chickens of each group were sacrificed on the 12th day after immunization. Single-cell suspensions derived from spleen in RPMI-1640 plus 10% FCS were prepared and loaded onto 6-well cell cultured plates (10^7 ^cells per well). The cells were incubated at 41°C in a 5% CO_2 _incubator. The cells were stimulated for 16 h with rYFP (5 μg/ml). Total RNA was extracted with Trizol^® ^(Invitrogen) and then transcripted using High Capacity cDNA Reverse Transcription Kit (Applied Biosystems). The primer pairs used for analysis of cDNA were showed in Table 1. Quantitative real-time PCR was performed on the 7500 Real Time PCR System (Applied Biosystems) with a program of 50°C for 2 min, 95°C for 10 min and 40 cycles of 95°C for 15 s; 60°C for 1 min. For each sample, template copy numbers were internally normalized with their respective input control. Relative expression was calculated as the ratio of template copy numbers of a sample relative to the naive control after normalizing to their respective isotype control Actin.

**Table 1:** Primer sequences used in Real-time RT-PCR

Sequence no	Gene name	Primer sequence
1	IFN-γ	F- CGCACATCAAACACATATCTG R- GATTCTCAAGTCGTTCATCGG
2	IL-4	F-AGGCAACACTACTTCAATGG R-GCTAGTTGGTGGAAGAAGGT
3	Actin	F-CCACACTTTCTACAATGAGCTG R-GGTCTCAAACATGATCTGTGTC

### Histological examination

The cecum of the challenged chickens was collected and fixed in 2.5% (v/v) glutaraldehyde-polyoxymethylene solution immediately after euthanization. The tissue samples were dehydrated and embedded in paraffin wax. Serial paraffin sections (4 um) were obtained and incubated at 37°C for at least 12 h. Paraffin was removed by three consecutive washings in xylol for 5 min each, and then hydrated with in 100, 95, 80, 70 and 50% alcohol and then deionized water. The histological paraffin sections were stained with Hematoxylin & Eosin (HE) and examined under a light microscope.

### Statistical analysis

The data were analyzed using the one-sided student's t-test. A P value less than 0.05 or 0.01 was considered significant.

## Results

### Transgenic *T. gondii *expressing the heterologous protein YFP

We engineered transgenic *T. gondii *expressing YFP as a model antigen by transfection of *T. gondii *RH with the plasmid pTgmicYFP containing a strong MIC2 promoter (Figure 1A). A stable transgenic line was cloned after several passages in mice and FACS sorting and limiting dilution. The exotic YFP protein expression was confirmed by western blotting. The transgenic *T. gondii *produced an expected band of 54.6 kDa protein recognized by anti-GFP antibodies, while no positive band was detected for the wild type *T. gondii *(Figure 1B). Fluorescence microscopy showed that *T. gondii *YFP expressed abundant YFP protein which was located in the cytoplasm of the parasites (Figure 1C). The transgenic *T. gondii *was co-passaged with the wild type *T. gondii *in mice and the proportion of the two lines remained constant after 4 passages (Figure 1D); this indicated that the fitness of the transgenic parasites was not altered after the integration of the exotic DNA into the parasite genome.

### Immunization with transgenic *T. gondii *YFP conferred partial protection against challenge with *E. tenella *YFP

**Figure 2:**
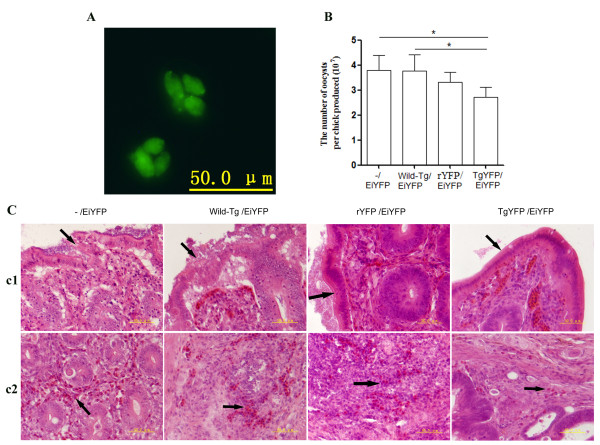
**The partial protection against challenge with *Eimeria tenella *YFP (EiYFP) in chickens which were immunized with *T. gondii***. Leghorn chickens immunized s.c. with two doses of *T gondii *YFP (TgYFP) or wild type RH strain (5 × 10^6 ^for the initial immunization and 10^7 ^for the booster dose) or 160 μg recombinant YFP emulsified in Freund complete adjuvant (FCA). The immunized chickens were challenged with 10^3 ^transgenic *E. tenella *YFP 15 days after the booster immunization. A. Fluorescence images of transgenic *E. tenella *YFP oocysts. B. Fecal oocyst counts in chickens immunized with *T. gondii *YFP (TgYFP) or wild type *T. gondii *tachyzoites (Wild-Tg) or recombinant YFP emulsified in FCA (rYFP) or complete cytomix buffer (un-immunized control) and challenged with the transgenic *E. tenella *(EiYFP). C. Histopathology of the cecum from chickens described above in Figure B. C_1 _Shedding of mucosal cells (arrow); C_2 _Inflammatory cells infiltration of lamina propria (arrow).

It has been shown that immunization of inbred mice with transgenic *T. gondii *expressing a foreign antigen of microorganisms can provide highly effective priming for CD8 T cell-dependent protective immunity against the microorganisms [1,18]. The YFP specific protective immune response was studied in chickens by immunization with *T. gondii *YFP followed by challenging with transgenic *E. tenella *YFP expressed in the cytoplasm (Figure 2A). Immunization with *T. gondii *YFP provided partial protection against *E. tenella *expressing the same antigen, YFP. Fecal oocyst output was significantly lower in the *T. gondii *YFP-immunized chickens than the wild type *T. gondii*-immunized or un-immunized chickens. The latter two groups yielded comparable number of oocysts. Compared with the rYFP immunized group, there was a decrease in fecal oocyst output in the *T. gondii *YFP-immunized chickens; although the small decrease in fecal oocyst output was not statistically significant (Figure 2B).

Histological examination of the cecum showed shedding of mucosal cells and inflammatory cell infiltration of the lamina propria in all groups, but the lesions in the transgenic *T gondii *group were less severe than the wild type and naïve groups and comparable to the rYFP immunized group (Figure 2C). These data suggest that the transgenic *T. gondii *YFP can elicit YFP specific immune responses in chickens. Previous studies showed that poultry are protected from coccidiosis mainly by cellular immunity [31,32]. The protection against *E. tenella *YFP infection in chickens observed in this study indicated that *T. gondii *YFP induced YFP specific cellular immunity.

### Humoral immune responses of chickens and rabbits to the transgenic *T. gondii*

**Figure 3:**
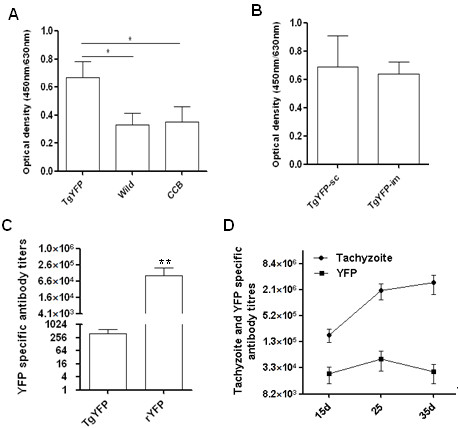
**Priming of AA broiler chickens and rabbits with *T. gondii *YFP (TgYFP) elicited antigen specific antibody responses**. A, YFP specific IgG in chicken sera (1: 25) 10 days after immunization by s.c. injection of 5 × 10^6 ^transgenic (TgYFP), wild type (WT) *T. gondii *tachyzoites or complete cytomix buffer (CCB). B, YFP specific IgG (1:25) 10 days after immunization by s.c. or i.m. injection. C, Serum YFP specific antibody titers in chickens 10 days after a s.c. injection of transgenic *T. gondii *tachyzoites (TgYFP) or i.m. injection of recombinant YFP (rYFP) protein expressed in *E. coli*. D, Kinetics of serum antibodies to YFP and tachyzoite antigens in rabbits immunized with the transgenic *T. gondii*. * P < 0.05, ** P < 0.01.

Here we investigated YFP specific humoral immune response in chickens and rabbits immunized with the transgenic *T. gondii*. AA broiler chickens were immunized s.c. or i.m. with 5 × 10^6 ^transgenic or wild type *T. gondii *tachyzoites (WT), 160 μg rYFP emulsified in FCA or CCB. Chickens primed with *T. gondii *YFP developed YFP-specific IgG, and the levels of anti-YFP IgG were significantly higher than those in chickens primed with wild type *T. gondii *or CCB (Figure 3A). Serum antibody titers in chickens injected i.m. with the transgenic parasites were comparable with the titers in chickens immunized by the s.c. route (Figure 3B). However, YFP specific antibody titers in chickens immunized with the transgenic parasites were markedly lower than those in chickens immunized with the rYFP protein (Figure 3C). Next, we investigated the humoral immune response in a susceptible animal model, the rabbit, to the transgenic *T. gondii*. Rabbits were immunized s.c. with 1 × 10^7 ^*T. gondii *YFP or WT strain, and serum antibodies to YFP and tachyzoite antigens were measured. A subcutaneous injection of the transgenic parasite elicited high levels of YFP specific antibodies, The IgG titers peaked on day 25 post immunization (Figure 3D). Compared with YFP specific antibodies, the host produced higher level of IgG to tachyzoite antigens (Figure 3D).

**Figure 4:**
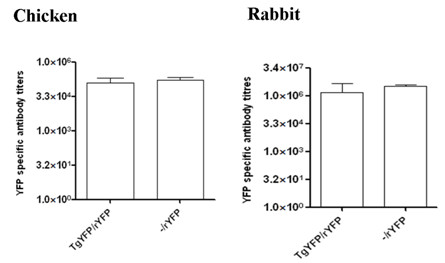
**Serum YFP specific antibody titers in chickens and rabbits immunized with *T. gondii *YFP and/or rYFP protein**. Chickens and rabbits were primed s.c. with 5 × 10^6 ^and 1 × 10^7 ^*T. gondii *YFP tachyzoites, respectively. The primed chickens were boosted i.m. 20 days later with 160 μg of rYFP emulsified in Freund complete adjuvant, and the primed rabbits were boosted i.m. 35 days later with 500 μg of recombinant YFP emulsified in Freund complete adjuvant. Serum YFP specific IgG titers were measured 10 days after the booster immunization.

Prior immunization with *T. gondii *YFP had no impact on humoral immune response to rYFP in either animal species. Similar serum YFP specific antibody titers were measured in chickens and rabbits injected with rYFP with or without prior immunization with *T. gondii *YFP (Figure 4). The above findings suggest that a subunit vaccine may be utilized to induce high level of humoral immune response in some animal species to make up for the low IgG titre induced by transgenic *T. gondii*.

### Transgenic *T. gondii *elicited YFP-specific cytokines expression

**Figure 5:**
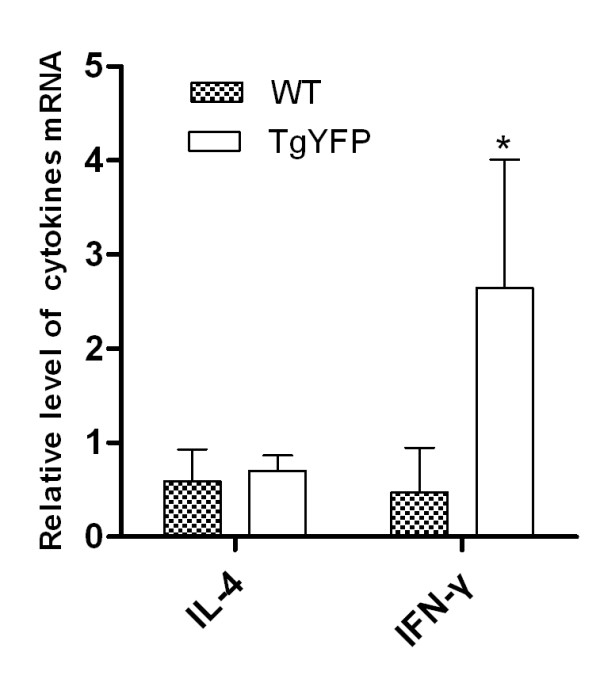
**IFN-g and IL-4 mRNA transcripts level of spleen lymphocytes**. The spleen lymphocytes were isolated on day 12 after immunization, and restimulated in culture for 16 h with rYFP, IFN-g and IL-4 mRNA transcripts level was measured by Real-time RT-PCR.

To further determine the YFP specific immune response induced by transgenic *T. gondii *YFP, we examined the level of IFN-g (Th1 type) and IL-4 (Th2 type) production by YFP specific T cells using Real-time RT-PCR. Single cell suspensions of lymphocytes were prepared from the spleen of the immunized chickens on day 12 after immunization, and restimulated in culture with rYFP. The results showed that the lymphocytes from transgenic *T. gondii *YFP immunized chickens produced significantly higher level of IFN-γ compared with wild type (WT) tachyzoites immunized group, while analysis of the IL-4 transcription revealed that there was no significant difference between the transgenic group (TgYFP) and the WT group (Figure 5).

## Discussion

Transgenic organisms expressing model antigens (e.g. ovalbumin, β-galactosidase) have been conveniently used to search for effective vaccine vectors. Protective effects of the model antigen specific immune response are evaluated by challenging the host with a different virus or bacteria strain or species expressing the same antigen [33]. Although some studies have already begun to explore the apicomplexan parasites as live vaccine vectors [34-38], there had been no studies to use transgenic parasites to express model antigen and induced immunity against infection by other transgenic Apicomplexa parasites, which have more complex life cycles and express more proteins than viruses and bacteria. Although both *Eimeria tenella *and *Toxoplasma gondii *are intracellular Apicomplexa parasites, there are significant differences in genome, proteome and life cycle [39,40]. Our study showed that the transgenic *T. gondii *expressing YFP induced immunity that partially protected chickens from the challenge with the transgenic *E. tenella *also expressing YFP. Therefore this study paved the way for the application of a heterologous challenge system to apicomplexa parasites.

Variations in susceptibility of different animal species to *T. gondii *infection are well known. The resistance is probably attributable to innate immunity or a rapid and strong immune response before the parasite can even disseminate. Chickens inoculated with *T. gondii *do not show any clinical symptoms [41], while rabbits are highly susceptible to *T. gondii *infection [42]. Both chickens and rabbits were chosen to evaluate the humoral immunity induced by the transgenic *T. gondii*. We observed that the YFP specific humoral immune response in the rabbit was significantly higher than that in the chicken. This implied that the susceptibility was one important determinant of the strength of immune responses. It was reported that six of seven *T. gondii *isolates from chickens were avirulent in mice, suggesting that the chicken was possibly more susceptible to avirulent *T. gondii *infection. Therefore, the avirulent *T. gondii *strain may be an effective vaccine vector for chickens.

It was reported that subunit vaccines predominantly induced high level neutralizing antibodies. To enhance the humoral immune response induced by the transgenic *T. gondii *in resistant animals, chickens immunized with *T. gondii *YFP were boosted with the YFP protein. The combined immunization regimen induced a high level humoral response in chickens compared to the weak humoral immunity induced by the transgenic parasite alone. Chicken whether or not they are vaccinated with *T. gondii *YFP prior to injection with rYFP produce the equivalent titers of anti-YFP antibody, this may be due to the superior ability of rYFP emulsified in FCA to induce high antibody titer which makes the effect of a boost is not visible. For the elicitation of strong cellular and humoral immune responses by transgenic *T. gondii*, the immunization with a combination of trangesnic parasites-based vaccine and a subunit vaccine should be considered.

Some intracellular pathogens have the coding capacity to produce distinct proteins that are sorted into different intracellular compartments [43,44]. Studies have shown that only a few proteins of *T. gondii *can stimulate CD8 T cell-dependent immunity in mice model [44,45]. Secreted, not cytoplasmic, antigens of *T. gondii *primed IFN-γ expressing CD8 T cells in mice which was susceptible to *T. gondii *infection [46]. Chicks, which were resistant to *T. gondii *infection, have many immunological mechanisms in common with mammals but have evolved distinct immunity to pathogens [47]. Here we showed that cytoplasm-localized YFP expressed by *T. gondii *can produce protective immune response in chicks that conferred partial protection against challenge with *E. tenella *YFP. To investigate whether this partial protection was related to the cytoplasm localization of YFP, a transgenic *T. gondii *line which secreted YFP need to be constructed in the future study to investigate the influences of antigen compartmentalization on the immune response in chickens.

## Conclusions

In conclusion, our study demonstrated the feasibility of using *T. gondii *as a delivery system to induce antigens specific protective immunity against the heterologous pathogen. The recombinant *T. gondii*-based vaccine priming and subunit vaccine boosting approach would be more effective than immunization with the transfected parasites alone in some animal species. The optimizations of *T. gondii *as a live vaccine vector to enhance the immune response need to be conducted in the future study.

## Competing interests

The authors declare that they have no competing interests.

## Authors' contributions

JZ, XXH, GWY, YD all participated in collecting the results presented here; HW and QJC contributed to the revision of the manuscript; XYL and XS supervised the study implementation and revised the manuscript. All authors read and approved the final manuscript.

## Authors' information

National Animal Protozoa Laboratory^a^, and Department of Veterinary Pathology^b^, College of Veterinary Medicine, China Agricultural University, Beijing, 100193, China. ^c ^Department of Etiology, Molecular Parasitology Laboratory, Institute of Basic Medical Sciences, Chinese Academy of Medical Sciences and Peking Union Medical College, Beijing 100005, China.

^d ^Institute of Pathogen Biology, Chinese Academy of Medical Sciences, Dong Dan San Tiao 9, Beijing 100730, China.
